# The C-terminal HRET sequence of Kv1.3 regulates gating rather than targeting of Kv1.3 to the plasma membrane

**DOI:** 10.1038/s41598-018-24159-8

**Published:** 2018-04-12

**Authors:** Orsolya Voros, Orsolya Szilagyi, András Balajthy, Sándor Somodi, Gyorgy Panyi, Péter Hajdu

**Affiliations:** 1 Department of Biophysics and Cell Biology, Faculty of Medicine, University of Debrecen, 400, 1 Egyetem sq., Debrecen, 4032 Hungary; 2 Department of Pediatrics, Faculty of Medicine, University of Debrecen, 400, 1 Egyetem sq., Debrecen, 4032 Hungary; 3 Department of Internal Medicine, Faculty of Medicine, University of Debrecen, 400, 1 Egyetem sq., Debrecen, 4032 Hungary; 4 Department of Biophysics and Cell Biology, Faculty of Medicine, University of Debrecen, 1 Egyetem sq., 4032, Hungary. MTA-DE-NAP B Ion Channel Structure-Function Research Group, RCMM, University of Debrecen, 400, Debrecen, Hungary; 5 Department of Biophysics and Cell Biology, Faculty of Dentistry, University of Debrecen, 400, 1 Egyetem sq., Debrecen, 4032 Hungary

## Abstract

Kv1.3 channels are expressed in several cell types including immune cells, such as T lymphocytes. The targeting of Kv1.3 to the plasma membrane is essential for T cell clonal expansion and assumed to be guided by the C-terminus of the channel. Using two point mutants of Kv1.3 with remarkably different features compared to the wild-type Kv1.3 (A413V and H399K having fast inactivation kinetics and tetraethylammonium-insensitivity, respectively) we showed that both Kv1.3 channel variants target to the membrane when the C-terminus was truncated right after the conserved HRET sequence and produce currents identical to those with a full-length C-terminus. The truncation before the HRET sequence (NOHRET channels) resulted in reduced membrane-targeting but non-functional phenotypes. NOHRET channels did not display gating currents, and coexpression with wild-type Kv1.3 did not rescue the NOHRET-A413V phenotype, no heteromeric current was observed. Interestingly, mutants of wild-type Kv1.3 lacking HRET(E) (deletion) or substituted with five alanines for the HRET(E) motif expressed current indistinguishable from the wild-type. These results demonstrate that the C-terminal region of Kv1.3 immediately proximal to the S6 helix is required for the activation gating and conduction, whereas the presence of the distal region of the C-terminus is not exclusively required for trafficking of Kv1.3 to the plasma membrane.

## Introduction

Potassium channels are essential players in setting the membrane potential and in the regulation of intracellular signaling in both excitable and non-excitable cells^1,2^. Voltage-gated potassium channels of the large family of K^+^ channels (Kv channels) are comprised of four subunits (both hetero- and homomers) in native cells and heterologous expression systems. A Kv channel subunit consists of six α-helical transmembrane segments (S1–S6). The intracellular N-terminal region of the channel contains the tetramerization T1 domain, which is required for assembly of individual subunits in the ER. Furthermore, accessory Kvβ subunits can bind to the N terminus, and enable the binding of several signaling molecules, such as kinases^3^. The highly-conserved pore region of Kv channels is formed by the linker between the S5 and S6, and mainly functions as a selectivity filter for K^+^ ions. The fourth transmembrane segment, which contains several positively charged amino acid residues, is considered to be the voltage sensor of all Kv channels^4^. The C-terminus of the channel can be coupled to various linker/adaptor proteins, which can anchor the protein to the cytoskeleton, bind to kinases or even regulate steering of the channels to the plasma membrane^5–10^.

Several studies have been published on the birth, membrane trafficking/targeting and assembly of channels^1,2^. During translation of the channel mRNA, the nascent polypeptide chain is embedded into the ER membrane, from which the balance between the retrograde and anterograde transport rates determines the expression level in the plasma membrane. Though many membrane proteins have a cleavable signaling sequence for targeting to the plasma membrane, Kv1 channels lack this motif and the S2 segment serves as a recognition site for targeting^1^. Other protein motifs were described in Kv1 channels that facilitate retention in the ER or forward targeting. For Kv1.4 channels the “VXXSL” motif of the intracellular C-terminus promotes high surface expression^11^. The pore region of Kv1.4 channels also governs targeting to the membrane. However, the Kv1.1 channel lacks the “VXXSL” sequence, instead it possesses the “HRET” amino-acid motif right after the S6 segment in the C-tail. Introduction of a stop codon after the R or H residues of this latter sequence leads to a loss of K^+^ conduction without altering the cell surface expression level^12^. Lu *et al*. showed that *Shaker* K^+^ channels, a Kv1 analogue in Drosophila, are also targeted to the plasma membrane without the “HRE” region of the C-terminal. The lack of the “HRE” region in *Shaker* resulted in a drastic change in the steady-state gating parameters^13^, as opposed to the loss of the conductance as in Kv1.1. On the contrary, deletion of amino acids preceding the “HRET” sequence in *Shaker*, (still part of the C-terminal region) prevented channel expression. Kv1.3 was shown to be guided to the cell membrane by the presence of two acidic glutamate (E) residues at the C-terminus of the channel: removal of these amino acids with C-terminal deletion or mutation to isoleucine resulted in the loss of ionic current and plasma membrane expression^14^.

Kv1.3 channels are present in various cell types including T cells, where they play an important role in controlling Ca^2+^ signaling upon antigen-induced activation. Two papers demonstrated recently that truncation of the Kv1.3 C-terminus can vastly deteriorate the anterograde transport of the channels^14,15^. It is well documented that the C-terminus of Kv channels is an important interaction site for several scaffolding proteins (PSD95, SAP-97, cortactin)^6,16–20^. Moreover, the “HRET” sequence in Kv1.3 channels, which is right after the S6 segment, is close to the activation gate^21^. Hence, truncation of the C-terminal including this motif may interfere with the activation gate and results in cell-surface targeting but a non-conducting construct. Consequently, the objective of this study was to clarify whether the removal of the major part of the C-terminal tail of Kv1.3 – with or without the HRET(E)-region – has any impact on the cell-surface trafficking or/and channel conductance of Kv1.3.

## Results

### Strategic considerations for designing the Kv1.3 constructs

**Figure 1:**
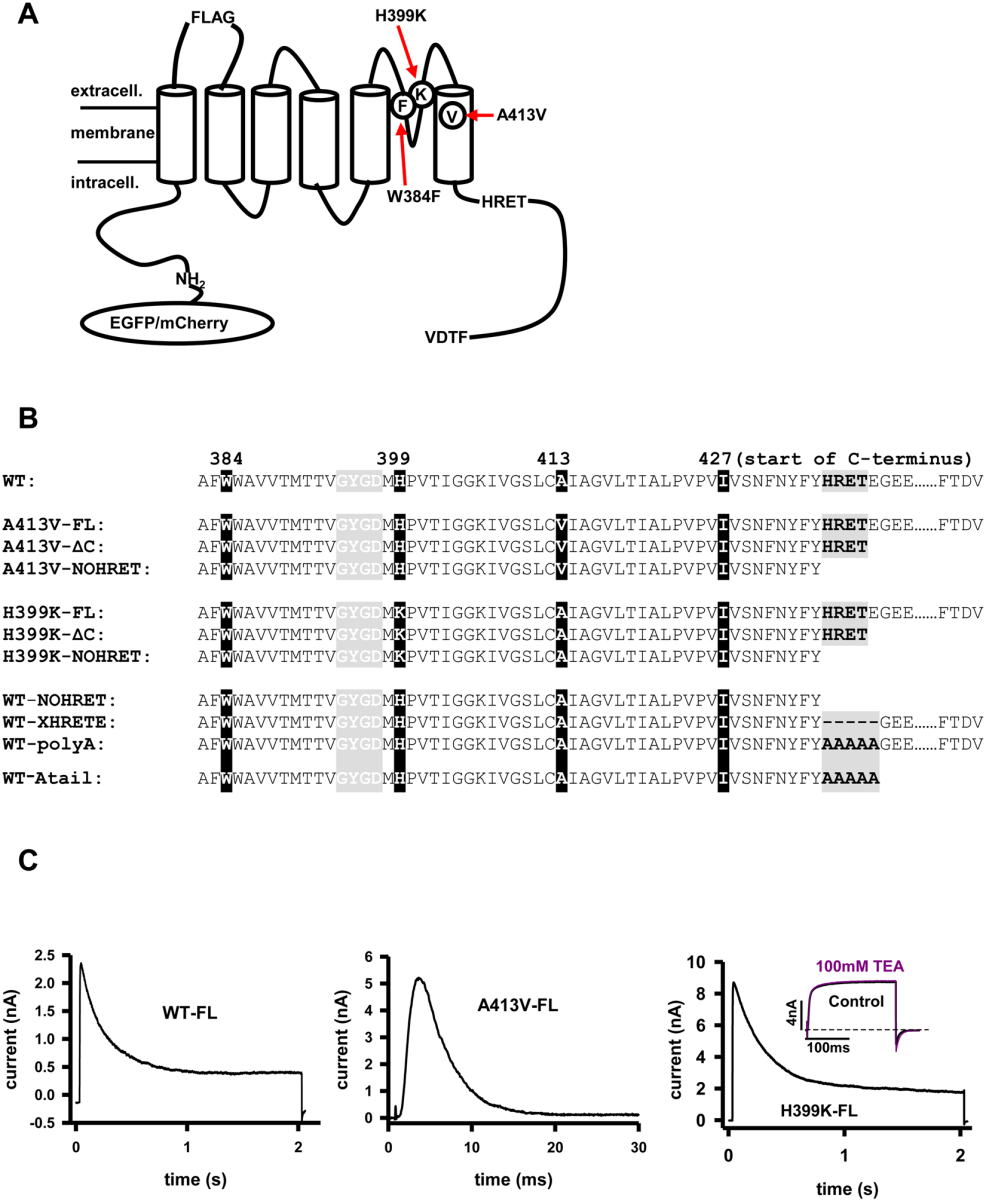
Point- and C-terminal deletion mutants of Kv1.3 channels. (**A)** Schematic illustration of wild type (WT) Kv1.3 pore-forming alpha subunit with N-terminal EGFP/mCherry, FLAG epitope, point mutations (H399K, A413V, W384F), the HRET sequence and the last four amino acids of C-terminus (FTDV). (**B)** Amino acid sequences of WT Kv1.3 and mutant Kv1.3 s starting at amino acid 382 (“……” represents rest of the C-terminus not relevant to this study). Amino acids are listed by their one letter designations, FL: full-length C-terminus, ΔC: C-terminal truncated at 439, NOHRET: truncation after postion 435. XHRETE: deletion mutant removing HRETE sequence. polyA: replacement HRETE with AAAAA, Atail: HRETE changed for AAAAA, and rest of C-terminus is removed. Starting amino acid of C-terminus 427 is indicated. (**C)** Current traces of WT (mCherry tagged, left), A413V-FL (center) and H399K-FL (right) channels expressed in HEK cells. WT (outside-out patch configuration): cells were depolarized to + 50 mV from a holding potential of −120 mV for 2 seconds. A413V-FL (whole-cell configuration): cells were kept at −120 mV then depolarized to + 50 mV for 30 ms. H399K (whole-cell configuration): cells were depolarized to + 50 mV from −120 mV for 2 seconds. Inset: whole-cell current of a cell expressing H399K-FL and depolarized to 0 mV from a holding potential of −120 mV for 200 ms in the presence and absence of 100 mM TEA.

Three strategies were applied for unique identification of the Kv1.3 subunits transfected into HEK or CHO cells. First, we introduced the A413V mutation in the S6, which has been shown to accelerate dramatically the inactivation kinetics of A413V homomers as compared to wild-type channels (τ_A413V_ = 4 ms, τ_WT_ = 200 ms, Fig. 1A and C^22,23^). As it was shown earlier, heterotetramers consisting of A413V and the WT subunits display intermediate inactivation kinetics between τ_A413V_ and τ_WT_ depending on the number of mutant subunits in the tetrameric channel^22,24,25^ (also see Suppl. Figure 1). Consequently, the presence of heteromeric channels can be easily tracked in the membrane by fitting the inactivation kinetics of the K^+^ current^23^.

Second, we substituted histidine (H) for lysine (K) at residue 399 which resulted in a TEA (tetraethylammonium)- insensitive, phenotype, as described earlier (Fig. 1A and C)^26–28^. The assembly of TEA sensitive (K_d_ ≈11.4 mM) and insensitive (K_d_≈2000 mM) Kv1.3 subunits modifies the affinity of the heterotetramer for TEA, hence the heteromultimer formation can be easily identified by the application of 100 mM TEA^26^.

Third, for immunocytochemistry experiments we used constructs that had the FLAG insert in the extracellular loop between the S1 and S2 segments. The insertion of the FLAG epitope does not alter dramatically the properties of Kv1.3 as shown earlier but allows labeling of the channels expressed in the plasma membrane.

These mutations were combined with various truncations/mutations in the C-terminus and fluorescence protein tagging on the N-terminus: EGFP for all constructs except the WT channel in the co-expression experiments, where mCherry was used. Truncations at the C-terminus were generated by introducing a stop codon after (constructs denoted with ΔC) or before the “HRET” sequence (labeled with NOHRET ending) (Fig. 1B).

### Deletion of the C-terminal region does not reduce current of ΔC channels

**Figure 2:**
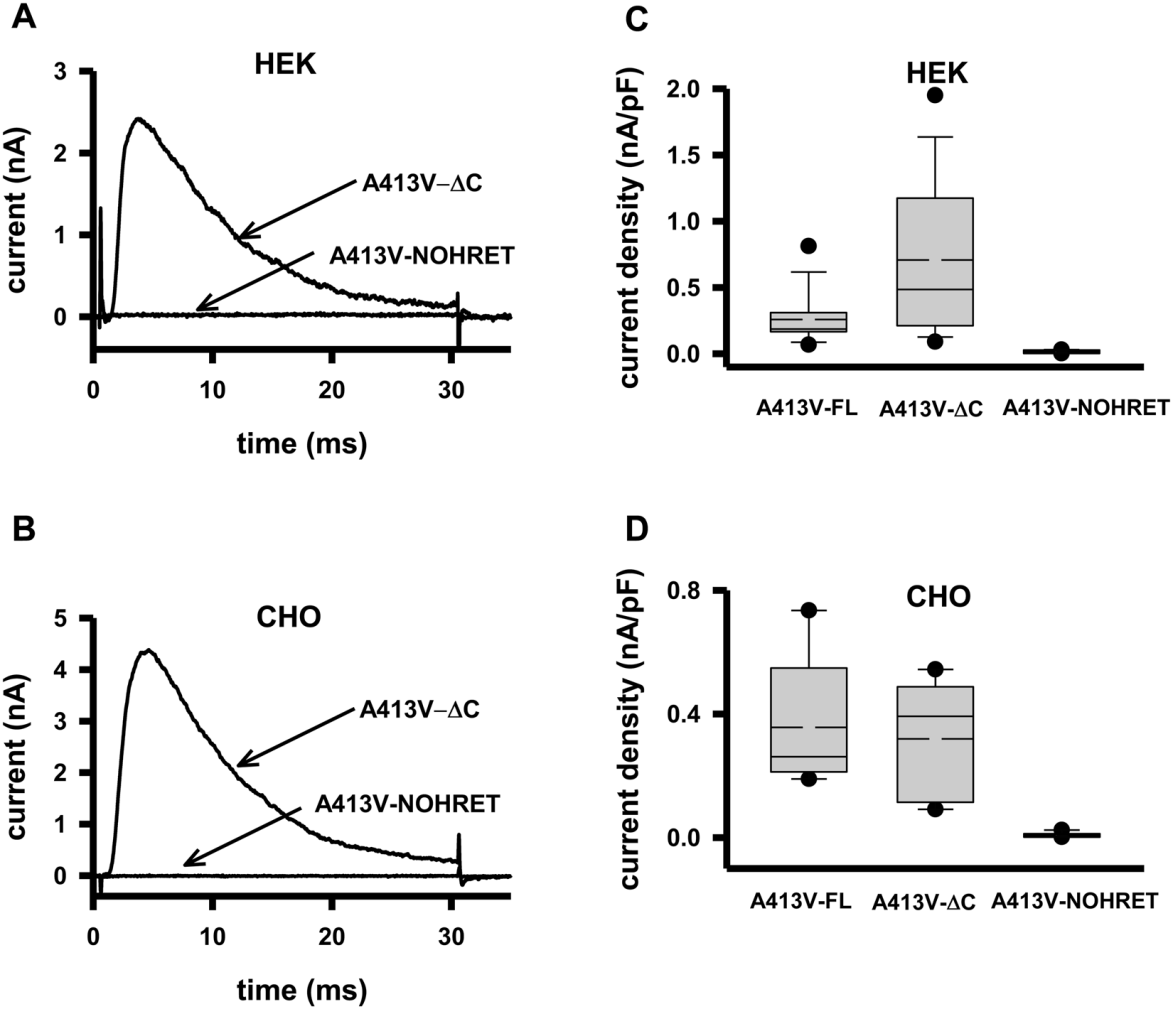
Current of A413V truncated mutants in HEK and CHO cells. **(A)** Representative current traces recorded in HEK transfected with the EGFP-tagged A413V-ΔC or the A413V-NOHRET construct (arrows). Currents were recorded upon 30-ms-long depolarizations to + 50 mV from a holding potential of −120 mV. **(B**) Representative current traces recorded in CHO transfected with the EGFP-tagged A413V-ΔC or the A413V-NOHRET construct. Recording conditions were the same as in panel A. **(C**,**D)** Box plot of the current density measured in HEK **(C)** or CHO **(D)** transfected with the full length (A413V-FL), A413V-ΔC or A413V-NOHRET constructs. The data are reported as mean (dashed line), median (solid line), first (top box) and third quartiles (bottom box), and maximum and minimum. Current density was determined as detailed in the materials and methods.

Several studies reported that the removal of the C-terminus of Kv channels drastically influences their cell membrane expression, and that the “HRET” sequence is crucial for anterograde trafficking^12^. To test this scenario for Kv1.3 we used the A413V-ΔC and A413V-NOHRET constructs (Fig. 1B) and expressed them in HEK and CHO cell lines. The ΔC nomenclature in this paper refers to a construct that is truncated right after the HRET motif (Δ440–523) whereas NOHRET indicates the construct lacks the entire C-terminus including the HRET motif as well (Δ436–523). Figure 2A shows that expression of the A413V-ΔC construct results in a robust whole-cell current having fast inactivation kinetics, as it was seen for full-length homotetrameric A413V (Fig. 1C). On the contrary, HEK transfected under identical conditions with the A413V-NOHRET construct did not show any voltage- and time-dependent current. Comparison of the current density (CD, peak current at + 50 mV divided by the whole-cell capacitance) for A413V-FL, A413V-ΔC and A413V-NOHRET in HEK cells (Fig. 2C) showed that the ΔC-truncated construct had a slightly higher median CD than the full-length one although the difference was not statistically significant (p = 0.053, CD_FL_ = 0.26 ± 0.05 nA/pF (n = 13, A413V-FL), CD_ΔC_ = 0.71 ± 0.15 nA/pF (n = 15, A413V-ΔC)) whereas the CD for the A413V-NOHRET is CD_NOHRET_ = 0.016 ± 0.007 nA/pF (n = 4), p < 0.05. To rule out the influence of the expression system on our results (e.g., the presence of endogenous Kv1.x channels/subunits in HEK, which may facilitate the forward trafficking of heteromers of WT and A413V-ΔC mutant subunits to the membrane, or cell-specific protein sorting and trafficking) the whole set of experiments was repeated in CHO cells^29,30^. CHO cells do not show measurable whole-cell outward current at + 50 mV (Suppl. Figure 2) and thus, are suitable for our experiments. The results in CHO were qualitatively similar to the ones obtained in HEK, the A413V-FL and the A413V-ΔC constructs gave rise to A413V homotetrameric currents of similar density whereas practically zero current could be recorded following the transfection with the A413V-NOHRET construct (Fig. 2D, CD_FL_ = 0.36 ± 0.1 nA/pF (n = 5, A413V-FL), CD_ΔC_ = 0.32 ± 0.09 nA/pF (n = 5, A413V-ΔC), p = 0.748, CD_NOHRET_ = 0.008 ± 0.002 nA/pF (n = 10), p < 0.05). Due to the lack of whole-cell currents in untransfected CHO we used these cells in our subsequent experiments.

We also tested whether all these channel constructs are properly translated by the cells using western-blot against a FLAG epitope inserted between the S1-S2 helical segments of all the constructs (Fig. 1A). Suppl. Figure 3 illustrates that CHO cells transfected with any of the three FLAG-tagged constructs express EGFP-tagged Kv1.3 channel subunit of appropriate size (between 85–95 kDa).

**Figure 3:**
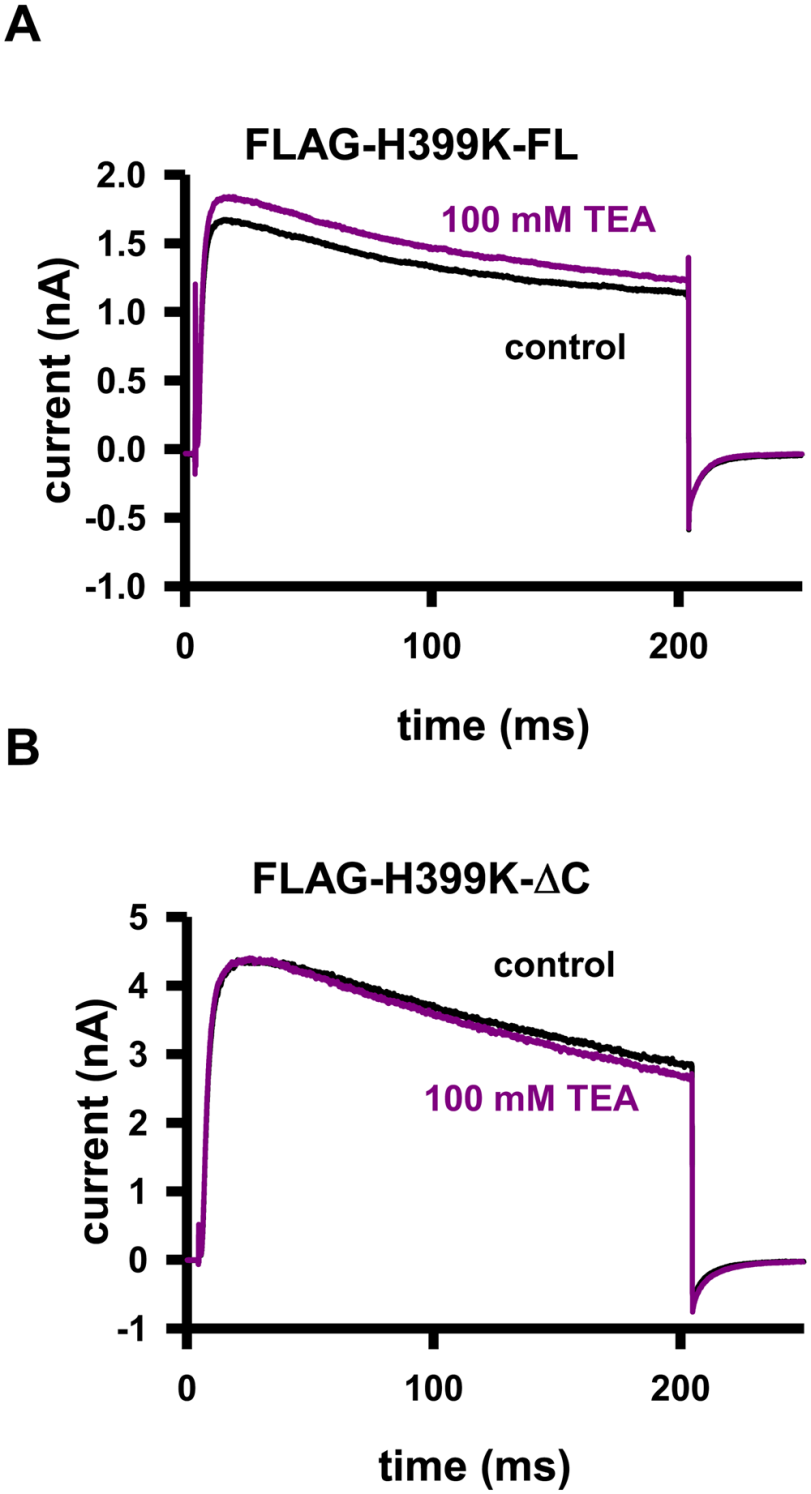
TEA inhibition of full length and C-terminal removed H399K channel. **(A)** Whole-cell currents of EGFP- and FLAG-tagged H399K-FL channels in CHO cells were recorded during 200-ms-long pulses to 0 mV (not to + 50 mV to reduce whole-cell current) from a holding potential of −120 mV. Traces are shown before (black) and after addition of 100 mM TEA (purple). **(B)** Currents of the EGFP- and FLAG-tagged H399K-ΔC construct in the presence (purple) and absence (black) of 100 mM TEA. The same voltage-clamp protocol was used as on panel A was used to evoke the currents.

### Pharmacology reveals homomers of H399K -ΔC channels in the membrane

To rule out mutation-specific effects (e.g. A413V) on the expression of the K^+^ current, we used an alternative, pharmacological approach to learn if carboxyl-terminal deleted channels can reach the plasma membrane. TEA, a general inhibitor of various K^+^ channels, is a fast open-channel blocker and its affinity depends on an aromatic amino-acid side chain in the extracellular mouth of the pore region^27,31^. Previously we showed that in Kv1.3 the mutation of the TEA-binding residue (H399) to a positively charged amino acid (e.g. lysine, K) led to a completely TEA-insensitive channel^26^. Figure 3A shows currents of the FLAG-H399K-FL (full-length subunits with FLAG tag) mutant in CHO cells in control extracellular solution and in the presence of 100 mM TEA, the overlapping currents indicate the lack of inhibition (RF = 0.95 ± 0.01, n = 5, see Materials and Methods). When CHO cells were transfected with the FLAG-H399K-ΔC plasmid we measured currents similar to the full-length H399K construct (Fig. 1C and Suppl. Figure 4A) and also displayed TEA resistance (Fig. 3B, RF = 0.98 ± 0.01, n = 5). The C-terminal deletion that includes the “HRET” motif as well (FLAG-H399K-NOHRET) resulted in a phenotype that lacks outward K^+^ current, just as we described for the A413V mutant (Suppl. Figure 4B).

In summary, using either kinetically tagged (A413V) or pharmacologically tagged (H399K) C-terminally truncated Kv1.3 we demonstrated that C-terminal truncated subunits (A413V-ΔC or H399K-ΔC) form functional homotetrameric channels in the membrane regardless of the expression system. However, we were unable to record whole-cell currents when the C-terminal truncated constructs that lack the “HRET” motif as well (i.e. A413V-NOHRET, H399K-NOHRET) were expressed either in HEK or CHO. This might mean impaired trafficking and/or conductivity/functionality of these latter constructs.

### NOHRET constructs can also target to the cell membrane

**Figure 4:**
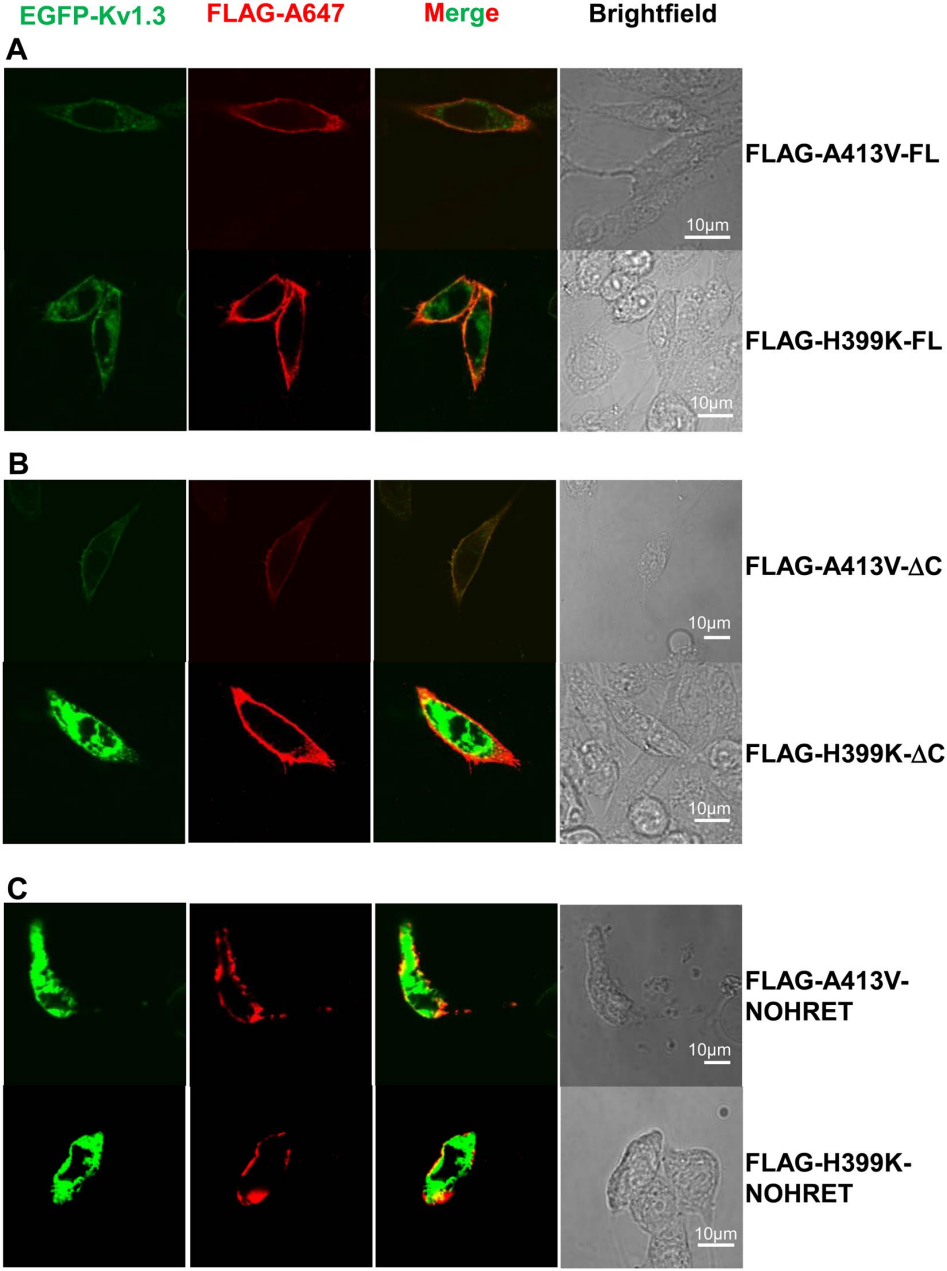
Targeting of deletion mutants of Kv1.3 to the plasma membrane. Representative images of EGFP- and FLAG-tagged Kv1.3 mutant channels expressed in CHO. **(A)** CHO cells expressing the A413V-FL/H399K-FL, **(B)** A413V/H399K C-terminal truncated Kv1.3 (A413V-ΔC/H399K-ΔC) or **C)** A413V-NOHRET/H399K-NOHRET plasmid is displayed. The 1^st^ column shows the EGFP signal of Kv1.3 channel in green. Cells were labeled with anti-FLAG primary and Alexa Fluor 647 GAMIG secondary antibodies to verify cell membrane localization of the channel protein (2^nd^ column, red). The 3^rd^ column shows the merge of red and green channels of the EGFP and anti-FLAG fluorescence signal whereas brightfield images of the cells are shown in the 4^th^ column. (For details of fluorescent labeling see *Materials and Methods*).

Next, we addressed if C-terminal truncated constructs that also lack the “HRET” motif can be expressed in the cell membrane. The Kv1.3 subunits were conjugated to EGFP on the intracellular N-terminus and the extracellular FLAG epitope was cloned into each plasmid encoding point and deletion mutants of the Kv1.3 channel (FLAG-H399K-FL, FLAG-H399K-ΔC, FLAG-H399K-NOHRET and FLAG-A413V-FL, FLAG-A413V-ΔC, FLAG-A413V-NOHRET constructs). The distribution of the channel subunits was detected by means of confocal fluorescence microscopy. Figure 4A shows that FLAG-tagged full-length, H399K and A413V mutant Kv1.3 channels were stained with anti-FLAG antibody in both CHO and HEK cells (only CHO is shown, for HEK see Suppl. Figure 5). The same was observed for both Kv1.3 point mutants that lacked either the complete C-terminus (ΔC, Fig. 4B) or the C-terminus plus the “HRET” motif (NOHRET, Fig. 4C). This confirms the presence of all of these constructs in the plasma membrane, though the intracellular retention of truncated channels was elevated as compared to the full-length counterpart as shown by the quantitative analysis of the images (Suppl. Figure 6). Moreover, no difference in membrane expression between the ΔC and the NOHRET constructs was detected for both A413V and H399K (Suppl. Figure 6). All these data support the hypothesis that Kv1.3 channels can reach the membrane even without the “HRET” motif and instead the gating/conductance of the channel is impaired.

**Figure 5:**
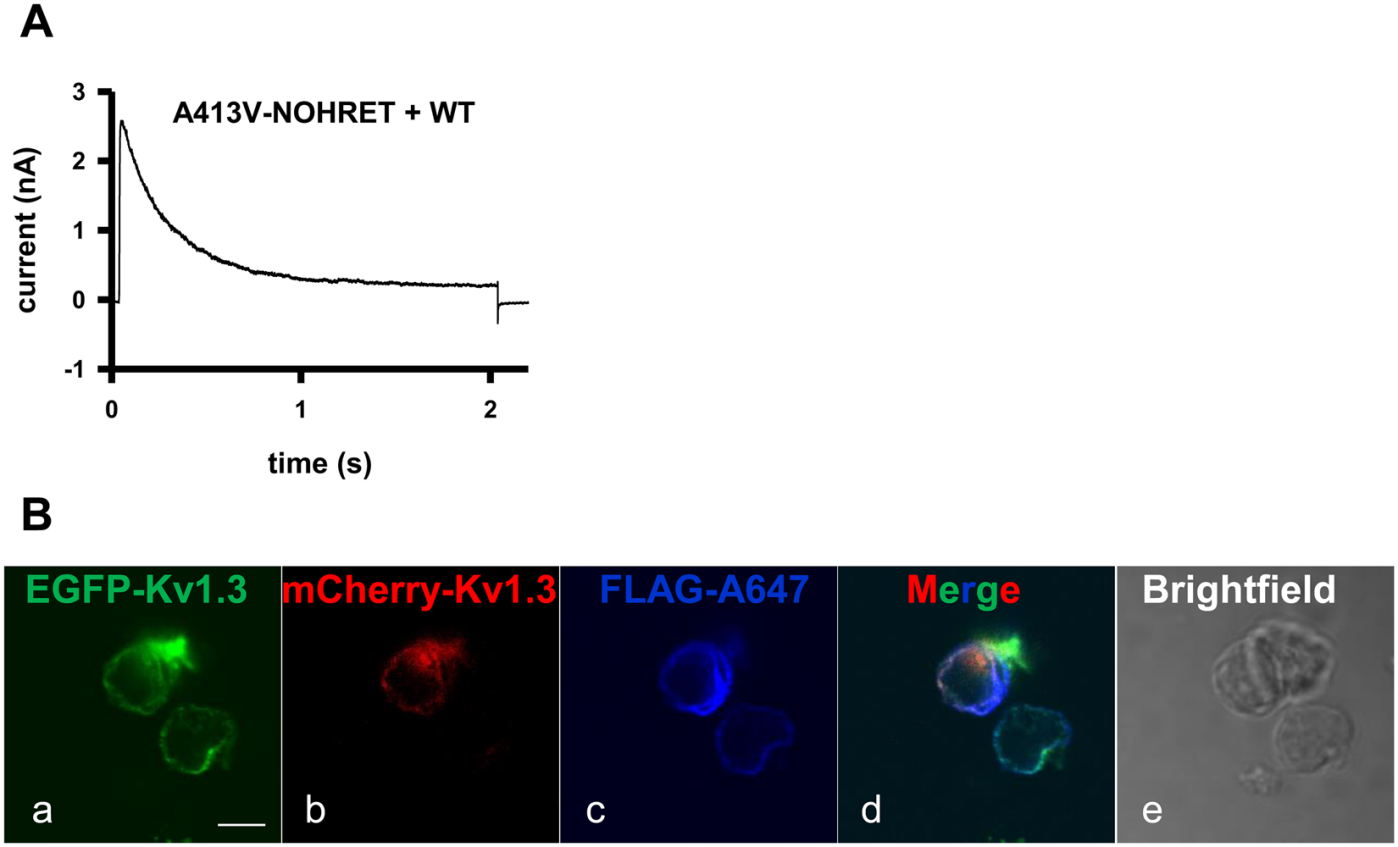
WT Kv1.3 fails to rescue the A413V phenotype when co-transfected with A413V-NOHRET subunits. **(A)** Whole-cell current recorded in a CHO cell co-transfected with EGFP-tagged A413V-NOHRET and mCherry tagged WT Kv1.3 plasmids (1:1 molar ratio). The cell was depolarized to + 50 mV from a holding potential of −120 mV. **(B)** Confocal images of a CHO cell expressing both FLAG and EGFP-tagged A413V-NOHRET and mCherry-tagged WT Kv1.3. Cells were stained with anti-FLAG and Alexa-647 GAMIG antibodies. *a:* A413V-NOHRET (green), *b*: mCherry-WT Kv1.3 (red), *c*: FLAG (blue), *d*: overlay of red, green blue channels, *e:* brightfield image of the cells. Scale bar is 5 µm.

**Figure 6:**
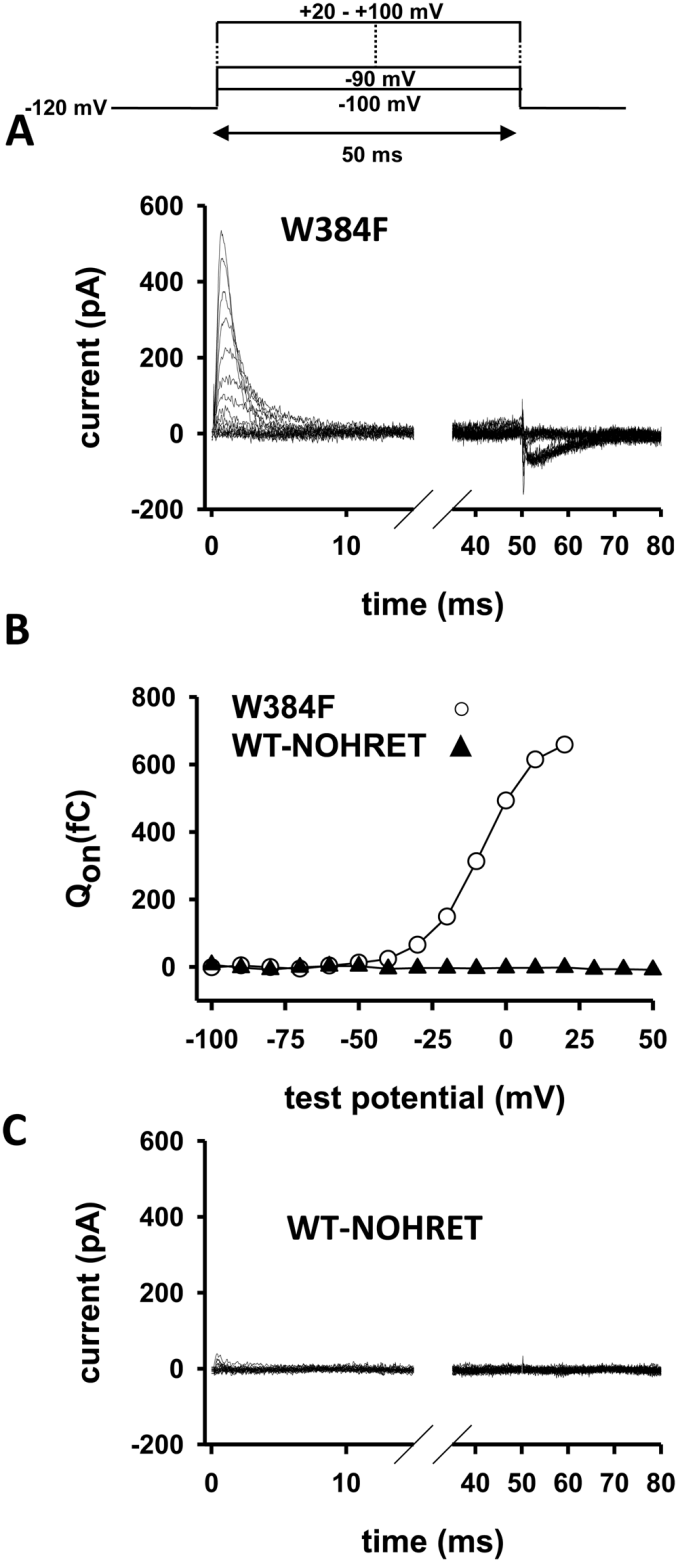
The gating current of WT-NOHRET channels is missing. **(A)** Gating currents in a WT-W384F channel expressing CHO cell. Cell was held at −120 mV then depolarized to different test potentials (from −100 mV to 20/100 mV in 10 mV steps) for 50 ms. **(B)** The test potential dependence of the integrated gating current (Q_on_, on-gating) for the cells recorded in panel A (W384F) and C (WT-NOHRET). Q_on_ was evaluated as described in the Material and Methods section. **(C)** Gating current recordings in a CHO cell transfected with WT-NOHRET construct. An identical pulse protocol was applied as in A.

### Co-expression of wild-type and A413V-NOHRET subunits results in pure wild-type Kv1.3 current

We co-transfected mCherry-tagged full-length, wild-type Kv1.3 channels along with EGFP-A413V-NOHRET plasmid mutants at 1:1 ratio and studied if the presence of the WT subunits can rescue conduction of the channels containing A413V-NOHRET subunits. Assuming that tetrameric channels assemble randomly from individual subunits a 1:1 co-transfection ratio should result in predominantly (87.5%) heteromeric channels having intermediate inactivation kinetics between WT and A413V^22^. On the contrary, we found that a CHO cell expressing both A413V-NOHRET and WT-Kv1.3 exhibits currents resembling the pure WT current (Fig. 5A); and not the “mixture” of multiple heteromeric currents characterized with various inactivation time constants (also see current trace of A413-FL and WT Kv1.3 in Fig. 1C and Suppl. Figure 1B). The current density of only WT-Kv1.3 channel expressing cells (3390 ± 1110 pA/pF) was far higher than those transfected with both WT-Kv1.3 and A413V-NOHRET constructs (1010 ± 311 pA/pF, p = 0.028). Moreover, we transfected CHO cells with a mixture of FLAG-tagged A413V-NOHRET (EGFP-conjugated) and WT-Kv1.3 (mCherry conjugated) and subjected them to anti-FLAG labeling as described in Fig. 4. As displayed in Fig. 5B the FLAG-epitope bearing A413V-NOHRET subunits are labelled by the anti-FLAG antibody, which indicates their membrane targeting.

### Gating charge movement of NOHRET channels is absent

To disclose if the conducting pathway or the activation gating is destroyed upon HRET removal in the NOHRET Kv1.3 we assessed the gating properties of WT-NOHRET construct expressed in CHO cells (see Fig. 1B). As a positive control, we expressed the WT-W384F channel, which is a non-conducting mutant of Kv1.3 (homologous to the non-conducting W434F mutant of the Shaker channel^32–37^). Figure 6A displays the gating currents recorded in a CHO cells stably expressing Kv1.3-W384F (we recorded gating currents in all 11 cells). The representative Q_on_-V curve for this cell in the Fig. 6B illustrates the sigmoid shape of membrane potential dependence of the integrated gating current, which is a hallmark of voltage-gated ion channels and point out the functionality of the voltage-sensor. When we measured the gating current in cells expressing WT-NOHRET channels no gating current was detected (n = 9, Fig. 6B and C) or a miniature gating current was detected at very depolarized test potentials of +50 mV or higher (n = 2, not shown). These indicate that voltage-sensor movement of the channel is compromised when HRET is not present.

### Deletion and substitution of the HRET(E) sequence does not affect the function of Kv1.3

**Figure 7:**
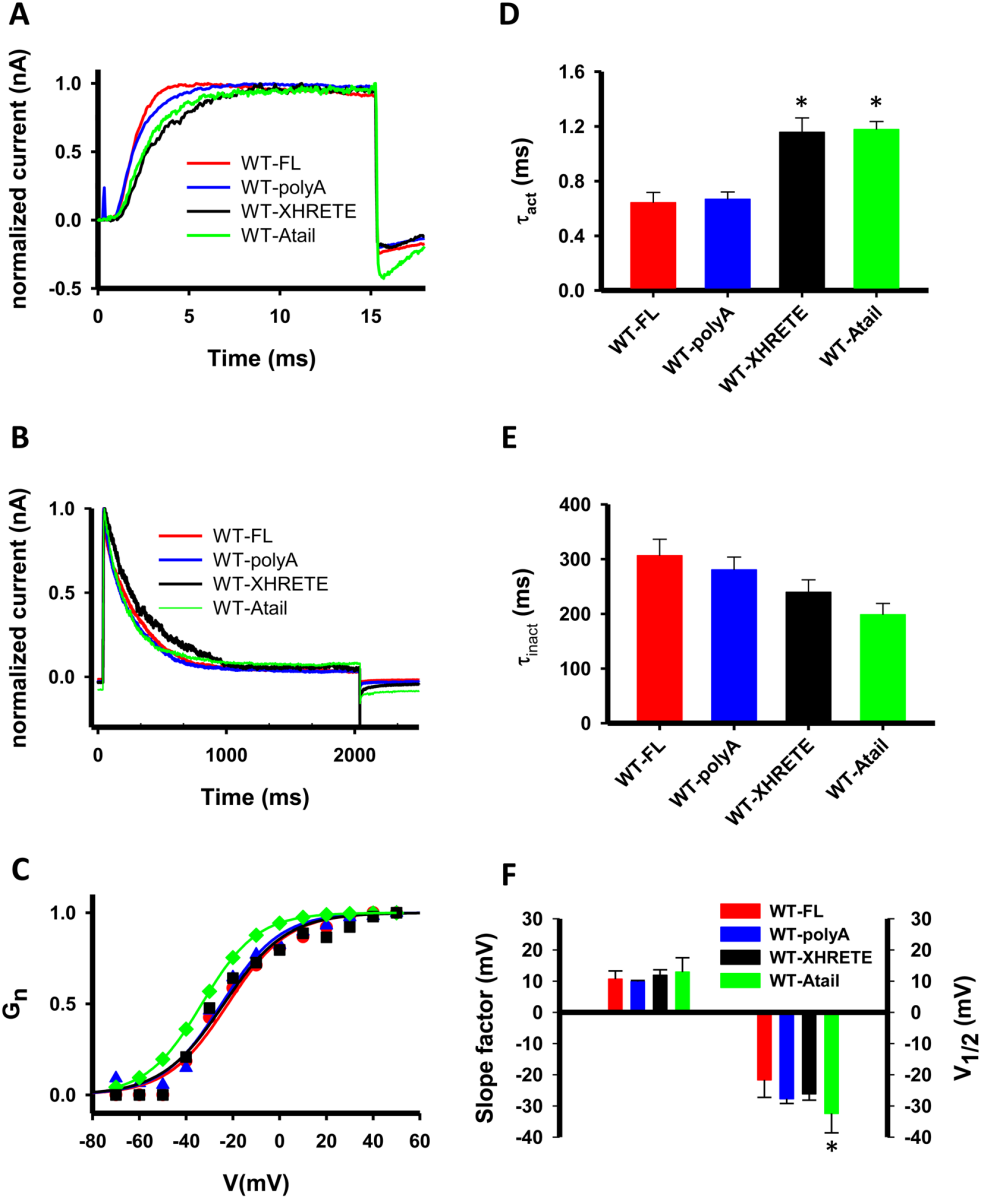
Elimination of the HRETE motif does not affect Kv1.3 operation. **(A)** Current traces of WT-FL, WT-XHRETE, WT-polyA and WT-Atail channels were recorded upon 15-ms-long + 50 mV depolarization to study activation kinetics of the four constructs (red line: WT-FL, blue: WT-polyA, black: WT-XHRETE, WT-Atail: green). For fitting and evaluation of activation kinetics see Materials and Methods. Holding potential was set to −120 mV, measurements were performed in outside-out configuration and currents were normalized to the peak value for the panel A and B. **(B)** Typical current recordings showing the inactivation kinetics of the WT-FL, WT-polyA, WT-XHRETE and WT-Atail channels. Cells were depolarized to + 40 mV for 2 s from −120 mV holding potential, inactivation kinetics was evaluated as described in Materials and Methods. **(C)** Voltage-dependence of steady-state activation of four Kv1.3 channel phenotypes: the normalized conductance-test potential relationships were obtained and analyzed as described in the *Materials and Methods*. Normalized conductance as a function of the test potential is shown for a WT-FL (filled circle, red line), a WT-polyA (empty triangles, blue line), a WT-XHRETE (empty square, black line) and WT-Atail (green diamonds, green line) channel expressing cell along with the best fit Boltzmann function. **(D–E)** The activation and inactivation time constants for the WT-FL, WT-polyA, WT-XHRETE and WT-Atail channels (mean ± SEM, n ≥ 6). Asterisk represents significant difference, p = 0.05. **(F)** Parameters of steady-state activation (slope factor (*k*), left and midpoint (*V*_1/2_), right) are displayed for the four constructs (mean ± SEM, n ≥ 6).

Motivated by these findings, mainly those for gating properties of NOHRET constructs, the following mutations were introduced into EGFP-tagged WT Kv1.3: 1) the extended HRET motif, HRET(E) region of the C-tail was deleted only (WT-XHRETE construct); 2) HRET(E) was replaced with a run of five alanines (WT-polyA channel) (see Fig. 1B, bottom sequences) and 3) the HRETE sequence was replaced with 5 alanines in the WT-∆C (WT-Atail channel). After transfecting these mutants and the WT full-length in CHOs we analyzed basic biophysical features for all channel types in outside-out patch configuration. As shown in Fig. 7, all three HRETE-manipulated subunits formed functional and conducting tetramers in the CHO cell membrane, and no major differences in the kinetic and equilibrium parameters of the gating were found. The activation kinetics was a bit slower for the WT-XHRETE and WT-Atail constructs as compared to WT-FL (p < 0.001 for both), but not the inactivation kinetics (p = 0.13). The half maximal voltage (V_½_) of the steady-state activation was only different for WT-Atail channels (p = 0.005, leftward shift) but the slope factor (k) was the same for all HRETE-mutants (WT-polyA, WT-Atail and WT-XHRETE, p = 0.284). Furthermore, we assessed the single-channel conductance for all four phenotypes and obtained that removal/replacement of HRETE-motif in Kv1.3 channels did not influence the unitary conductance (Suppl. Figure 7, p = 0.085). Moreover, FLAG-epitope bearing WT-Atail channels could be detected on the cell surface (Suppl. Figure 8). All these data clearly demonstrate that the HRETE motif is not vital for the operation of Kv1.3: the channel is present in the plasma membrane, and it is functional in the absence of this motif.

## Discussion

Membrane targeting of Kv channels has been studied by multiple groups, as it is a key player in the regulation of various cellular processes including action potential regulation or immune response^38^. To draw appropriate conclusion for trafficking it is critically important to demonstrate unambiguously that the current recorded is a consequence of the ion channel genes transfected into the cells and not influenced by endogenous K^+^ channel subunits in the expression system. To do this we used kinetically or pharmacologically tagged Kv1.3 subunits with properties that uniquely distinguish homotetrameric channels formed by transfected subunits from endogenous K^+^ channels or hetermultimers of endogenous and transfected channel subunits. If the A413V-ΔC subunits reach the plasma membrane because they form heterotetramers with endogenous WT Kv1.3 in HEK cells, then the inactivation kinetics of the whole-cell current should be fitted with sum of multiple exponential decay functions (see Suppl. Figure 1). On the contrary, we obtained that τ_i_ for A413V-FL (4.1 ± 0.6 ms) was the same as that of the A413V-ΔC (5.7 ± 0.5 ms, p = 0.15), and the decaying part of the curves could be fitted with a single-exponential function (data not shown). The expression of A413V-ΔC current in CHO cells, which do not exhibit any voltage-gated K^+^ conductance, supports the scenario as well that ΔC-truncated mutants are targeted to the plasma membrane without their combination with full-length Kv1.x subunits.

The outcomes with the H399K mutant also support the hypothesis that C-terminus removal downstream of the “HRET” region does not prevent targeting of Kv1.3 to the cell membrane. For H399K-ΔC and H399K-FL the inhibition by 100 mM TEA was negligible, which verifies that there was no mixing of H399K-ΔC and WT Kv1.3 subunits in the ER (we used CHO cells here as well, which have no Kv1 subunits), otherwise we should have seen block by TEA^27,31^. In addition, we detected the FLAG epitopes of both A413V-ΔC and H399K-ΔC subunits in transfected cells (CHO and HEK) with immunocytochemistry in non-permeabilized cells, which serves as an additional proof for their plasma membrane localization (Fig. 4 and Suppl. Figure 5) Interestingly, we also found that A413V-NOHRET or H399K-NOHRET bearing the FLAG-epitope showed a membrane signal upon anti-FLAG staining, although patch-clamp experiments revealed that these deletion mutants produced no current.

Our results somewhat contradict the findings of previous studies which emphasized the importance of the C-terminal region downstream of the “HRET” motif in Kv1.3, Kv1.2 and Kv1.1 in regulating trafficking^14,15^. The importance of a diacidic signal (E483/484) in the C-terminal region of Kv1.3 was suggested to control surface expression via interaction with Sec24 (a coat protein of vesicular transport)^14^. Another study demonstrated that alanine-scanning mutational analysis of glutamates at residues 438/440/442 (in our construct) slightly increased ER retention only for triple mutants, and Sec24a and Sec24b knock-down inhibited from-ER-to-Golgi transport^15^. Furthermore, the removal of a much shorter fraction of the C-terminus, as compared to our study, was sufficient to cease the anterograde transport of Kv1.3^14^. On the contrary, Kv1.3 in our hands is targeted to the plasma membrane in the absence of the entire C-terminus that includes the HRET sequence, however, increase in the intracellular retention of the channel protein was observed in the truncated channels. (Suppl. Figure 6, Fig. 4). We suppose that the use of different Kv1.3 genes (human in this study vs rat and mouse in others) cannot explain the difference due to multiple reasons: 1) we have reported previously that various C-truncated rat Kv1.3 channels had high membrane expression^16^, 2) the mouse, rat and human Kv1.3 genes have high homology, 3) in our recent paper we showed that the C-terminal deletion of WT hKv1.3 (stop codon after HRET motif) results in the expression of currents comparable to that of the full-length WT Kv1.3^10^. We think that the lack of or the very low expression detected by others for the C-terminal truncated constructs can be attributed to the cell lines used to express these constructs^12,14,29^. For example, glycosylation or other post-translational modifications can modify the surface expression level of Kv1.x channels; or the heteromerization of other Kv subunits that are endogenously present in these cells with Kv1.3 may prevent the trafficking to the plasma membrane^29,39–45^. Alternatively, the mutations cited above may create a retention signal that affect the trafficking of the channels to the membrane. As shown here, we ruled out cell line specific conclusions by using two cell lines and mutation specific effects by using two point mutants having unique characteristics.

On the other hand, our results are in harmony with others showing that the lack or modification of the HRET sequence does not terminate cell surface expression of Kv1.1 or the *Shaker* channel without the equivalent “HRE” sequence makes it to the membrane, similar to Kv1.3 in this study^12,13^. In addition to trafficking, the role of the HRET(E) sequence was suggested in regulating the conductance of various channels. For example, the arginine in the HRET(E) sequence of Kv1.1 is critical to form a conductive channel and the Shaker channel lacking the HRE sequence shows altered steady-state activation gating^12,13^. Our results partially agree with these studies: the C-terminally truncated Kv1.3 that lacks the HRET sequence is expressed in the surface membrane but does not conduct K^+^ current.

However, we went much further in understanding this phenomenon. First, WT Kv1.3 could not rescue the conductance of the A413V-NOHRET subunits in CHO cells, i.e., co-transfection of these two subunits resulted in pure homotetrameric WT currents. Fluorescence signals clearly showed the presence of both subunits in the plasma membrane, thus, interpretation of these results is that either WT Kv1.3 and A413V-NOHRET subunits do not form heterotetramers or that the presence of A413V-NOHRET subunits in a heteroteramer renders the channel non-conductive in a dominant negative manner. The fact that cells transfected with WT-Kv1.3 had much greater current density as compared to cells expressing both constructs support the formation of non-conducting heterotetramers in the cell membrane (i.e., some WT subunits are engaged in non-conducting heterotetramers). As tetramerization of Kv1.3 is governed by the N-terminal tetramerization domain, rather than the C-terminus, we favor the dominant-negative effect of the mutant subunits (as has been demonstrated for other mutations as well)^1,46–48^.

The HRET(E) is sequence is located in the part of the C-terminal that is proximal to the activation gate or may be part of it^49^. Thus, mutation in this region may profoundly alter activation gating of the channels, the coupling of the voltage-sensor movement to the activation gate or both while leaving the voltage-sensor movement intact. To test this assumption, we determined the gating currents of WT-NOHRET channels. To our surprise, we found that majority of the cells expressing this channel did not exhibit gating currents that resemble the gating current of the non-conducting W384F mutant (Fig. 6), which was used as a control. As fluorescence signals confirm the surface expression of the NOHRET construct this result suggests that voltage-sensor movement is impaired in the NOHRET Kv1.3 that lacks the full C-terminus including the HRET sequence. The origin of this unknown “reverse coupling” (i.e. movement of the voltage sensor is impaired by modification of the activation gate region) is unknown, but it does not seem to be specific for the HRET sequence. When we deleted just the HRETE sequence (WT-XHRETE construct) or substituted with alanines (WT-polyA) and left the rest of the carboxyl-terminus intact, or changed HRETE for five alanines in the WT-Atail construct (and rest of C-terminus was removed) the conductance of Kv1.3 was recovered. So it seems that the lack of a peptide strand on the C-terminus at the activation gate renders the channels non-conductive and any replacement may substitute for the HRETE sequence, at least in Kv1.3.

In summary, we demonstrated that Kv1.3 channel trafficking to the plasma membrane is preserved even if the whole C-terminus, including the HRET sequence is deleted. This finding highlights that trafficking motifs may not be universal and their importance must be tested for each channel/expression system combination. A similar conclusion can be drawn for the role of the HRET sequence in regulating channel conductance^12,49^: in case of Kv1.3 even an alanine substitution of the HRETE sequence restored channel function. Based on this and other recent papers the presence of the C-terminal amino acids adjacent to the activation gate in Kv1.3 are important for maintaining ion conductance, whereas distant C-terminal amino acids govern interactions with other proteins or confer cholesterol sensitivity to the Kv1.3^50^.

## Materials and Methods

### Kv1.3 channel constructs

The wild-type human Kv1.3 channel (shorter version of Kv1.3, composed of 523 amino acids acc. #: M85217^51^) was subcloned into pEGFP-C1 plasmid (Clontech, USA) using Bgl II and EcoR I restriction sites (Thermofisher, Hungary). For sticky-end ligation, T4 DNA ligase (Thermofisher, Hungary) was used according to the standard protocol. The deletion (ΔC, NOHRET, XHRETE) and insertion/point (FLAG tag, polyA, Atail, W384F) mutants were generated with flanking-primer mutagenesis method. Point-mutant (H399K, A413V), full-length constructs in pRc-CMV were made before as described in^22,25^. For co-transfection of wild type (WT) and mutant Kv1.3 plasmids mCherry tagged WT Kv1.3 was generated in two steps with PCR-based cloning: first EGFP was replaced with mCherry in pEGFP-C1 plasmid, then the wild-type Kv1.3 gene was subcloned into this pmCherry-C1 plasmid with EcoR I and Bgl II enzymes. All constructs were sequenced at the Clinical Genomics Center at University of Debrecen.

### Cell culture and transfection

HEK-derived tsA-201 (later we call them HEK for simplicity) and CHO (chinese hamster ovary, both from ATCC, Germany) cells were cultured in DMEM medium (Sigma-Aldrich Ltd., Hungary), which contained 10% FBS, 1 mM Na-pyruvate, and 200 units penicillin/streptomycin. Cells were maintained at 37 °C in a humidified atmosphere of 5% of CO_2_ and 95% air. Cells were passaged every 2–3 days. Transfections of DNA plasmids were performed using Lipofectamine 2000^TM^ (Life Technologies, Hungary) according to the manufacturer’s protocol. The patch-clamp and immunocytochemistry experiments were performed 24 hours post transfection. The CHO cell line stably expressing EGFP-tagged W384F-Kv1.3 was established as detailed in ref.^10^.

### Electrophysiology

HEK and CHO cells transfected with various Kv1.3 channel constructs were washed with standard extracellular solution (composition was in mM: 145 NaCl, 5 KCl, 1 MgCl_2_, 2.5 CaCl_2_, 5.5 glucose, 10 HEPES, pH: 7.35), and plated onto cell culture 35-mm petri dishes. Kv1.3 currents were recorded in whole-cell and outside-out configuration using an Axopatch 200B amplifier (Molecular Devices, Sunnyvale, CA, USA) as previously described[]^52^. The pipette filling solution consisted of (in mM): 140 KF, 11 K_2_EGTA, 1 CaCl_2_, 2 MgCl_2_, and 10 HEPES (pH 7.20, ~295 mOsm). TEA (tetraethylammonium) containing bath solution contained (in mM): 100 TEA-Cl, 45 NaCl, 5 KCl, 1 MgCl_2_, 2.5 CaCl_2_, 5.5 glucose, 10 HEPES (pH 7.35, 305 mOsm). For gating-current and single-channel measurement the external bath did not contain Na^+^ and the composition was the following (in mM): 145 choline-Cl, 5 KCl, 1 MgCl_2_, 2.5 CaCl_2_, 5.5 glucose, 10 HEPES (pH 7.35, 305 mOsm) (Na^+^ had to be omitted from the solution since some CHO cells expressed inward Na^+^ current). P/5 protocol for online leak subtraction was applied to minimize capacitance and leak errors during the measurements with the A413V mutant. Perfusion of the cell with different test solutions was achieved using a gravity-flow perfusion setup, and excess fluid was removed continuously.

The current density was defined as the ratio of peak current detected at +50 mV test potential and the whole-cell capacitance (read from the compensatory circuit of the amplifier). The remaining fraction of the current (RF) for TEA inhibition was defined as the ratio of the peak current measured after and before perfusion with 100 mM TEA.

The activation kinetics of the current was characterized by fitting the Hodgkin-Huxley (HH) model (I(t) = I_a_ × (1−exp(−t/τ_a_))^4^ + C where I_a_ is the amplitude of the activating current component; τ_a_ is the activation time constant of the current; C: constant) to the rising phase of the current traces evoked by 15-ms-long depolarizations to +50 mV. The activation time constant characteristic of a given cell was determined as the average of the time constants obtained upon three sequential depolarizations repeated every 15 s. P/5 protocol for online leak subtraction was applied.

The inactivation kinetics of the current was characterized by fitting a single exponential function (I(t) = I_0_ × exp(−t/τ_in_) + C, I_0_: amplitude of current, τ_in_: inactivation time constant, C: steady-state value of whole-cell current at the end of the pulse) to the decaying part of the current traces evoked by 2-s-long depolarizations to +40 mV from a holding potential of −120 mV. The inactivation time constant for a given cell was determined as for τ_a_, except pulses were delivered every 60 s.

The voltage-dependence of steady-state activation relationships were obtained as follows. The cells were held at −120 mV holding potential and depolarized to various test potentials ranging from −70 up to + 50 mV in 10 mV steps at every 30 s. Peak whole-cell conductance (G(*V*)) at each test potential was calculated from the peak current (I_p_) at test potential *V* and the K^+^ reversal potential (E_r_ = −85 mV) using G(*V*) = I_p_/(*V*−E_r_). The G(*V*) values were normalized for the maximum conductance and plotted as a function of test potential and the Boltzmann-function was fitted to the data points: G_N_ = 1/(1 + exp [− (*V*−*V*_½_)/*k*], where G_N_ is the normalized conductance, *V* is the test potential, *V*_½_ is the midpoint and *k* is the slope of the function.

Gating currents were determined using a non-conducting Kv1.3 mutant (W384F-Kv1.3). A voltage step protocol was applied from −100 mV up to 20–100 mV with an increment of 10 mV, each step lasted for 50 ms, P/5 protocol was used for leak subtraction (pulses were opposite to the test potential) to reduce capacitance and leak errors during the measurements. The gating charge was calculated upon the integration of the area under gating current traces.

### Immunocytochemistry

For Fig. 5 and Suppl. Figure 5: HEK and CHO cells expressing FLAG bearing EGFP-Kv1.3 plasmids were plated onto poly-L-lysine coverslips and incubated for 1 hour (37 °C, humidified, 5% CO_2_). Then cells were fixed with 1% formaldehyde and labeled with mouse anti-FLAG M2 antibody (1:1000, Sigma-Aldrich Ltd., Hungary). Secondary antibodies (goat anti-mouse with Alexa647, Thermofisher, Hungary) were added to the cells for 1 hour. Finally, coverslips were mounted onto slides with Fluoromount G (eBioScience, USA). Zeiss LSM 510 META and Olympus FV-1000 microscopes were used to take confocal images of the cells. A He-Ne laser was selected to excite Alexa647 and mCherry (line 633 and 543 nm) and an Argon laser (line 488 nm) to visualize EGFP. The thickness of the slices was set to 1 µm.

For Fig. 4. and Suppl. Figure 8: CHO cells were plated into 8-well chamber and transfected with FLAG bearing EGFP-Kv1.3 plasmids (37 °C, humidified, 5% CO_2_). After 24 hours the cells were washed and then fixed with 1% formaldehyde and labeled with mouse anti-FLAG M2 antibody for 2 hours, 37 °C (1:500, Sigma-Aldrich Ltd., Hungary). Secondary antibodies (goat anti-mouse with Alexa647, 1:500, Thermofisher, Hungary) were added to the cells for 1 hour. Zeiss LSM 510 META and Olympus FV-1000 microscopes were used to take confocal images of the cells. A He-Ne laser was selected to excite Alexa647 (line 633 nm) and an Argon laser (line 488 nm) to visualize EGFP. The thickness of the slices was set to 1 µm.

### Western blotting

Protein samples were separated by SDS-PAGE and transferred to PVDF membranes (Millipore, Billerica, MA, USA) after electrophoresis. The membranes were blocked with milk powder, and immunoblotted with mouse-anti-FLAG M2 (Sigma-Aldrich Ltd., Hungary) or rabbit-anti-actin (Sigma-Aldrich Ltd., Hungary) primary and anti-mouse IgG HRP-linked or anti-rabbit IgG HRP-linked secondary antibodies (Cell Signaling Technology, Inc., Beverly, MA), respectively. Blots were developed with ECL reagent (Thermo Scientific Inc., Vantaa, Finland). The blots were then visualized with the FluorChem Q MultiImage III Western blot imaging system (ProteinSimple, USA).

### Statistical analysis

Data are reported as the mean ± standard error. Means were compared using Student’s t-test or one-way ANOVA. P-value was set to 0.05. Statistical analyses were performed using SigmaPlot version 10.0 (Systat Software Inc.).

### Reagents

All reagents were purchased from Sigma-Aldrich (St. Louis, MO, USA), unless stated otherwise.

### Data availability statement

Data published in this study available upon reasonable request from the corresponding author.

## Electronic supplementary material

Supplementary information
