# Supplementary material for: The C-terminal HRET sequence of Kv1.3 regulates gating rather than targeting of Kv1.3 to the plasma membrane
Source: Sci Rep. 2018 Apr 12;8:5937. doi: 10.1038/s41598-018-24159-8 (PMC5897520; doi:10.1038/s41598-018-24159-8)
Supplement: Supplementary file 1 — Supplementary information [file 41598_2018_24159_MOESM1_ESM.pdf]

The C-terminal HRET sequence of Kv1.3 regulates gating rather than targeting of Kv1.3 to the plasma membrane

Orsolya Vörös, Orsolya Szilagyi, András Balajthy, Sándor Somodi, Gyorgy Panyi, Péter Hajdu

Supplementary Information

Supplementary figure 1. Assembly of WT and A413V subunits. A) The homotetrameric and heterotetrameric channels formed by WT and A413V subunits upon co-transfection. Heterotetramers of WT and A413V subunits make up a current with τ_i of 11 ms (3 A413V and 1 WT subunit), 28 ms (2-2 A413V and WT) and 75 ms (1 A413V and 3 WT subunits) (here we assumed that $\tau_{i,0}$=200 ms for WT homomers and $\tau_{i,4}$=4 ms for A413V homomers as in, equation is shown on panel C, bottom equation). The assembly of subunits is random, the ratio of the channel species is determined by the binomial distribution (see panel C, top equation, first term). **B)** Normalized whole-cell current trace of the mixture of homomeric and heteromeric channels recorded upon the co-transfection of a CTLL cell (a mouse T cell line) with WT and A413V Kv1.3 (+50 mV depolarization from a holding of -120 mV). The best-fit line was fitted using the cooperative model shown on panel C [1]. **C)** The normalized current was fitted with the top equation assuming 5 channel species built up from WT and A413V subunits. I_{peak}: peak current; m: number of mutant subunits in a channel species (from 0 up to 4); p: fraction of the mutant subunits; R: ratio of the steady-state current at the end of the pulse and the peak current (I_{peak}).

Supplementary figure 2. No outward current in the untransfected CHO cells. Typical current trace was recorded in a CHO cell upon 30-ms-long, +50 mV depolarization from a holding potential of -120 mV.

Supplementary figure 3. Western blot of total cell lysates using specific FLAG and actin antibodies. CHO (A) and HEK/CHO (B) cells were transfected with FLAG-bearing A413V-FL,

A413V-ΔC or A413V-NOHRET plasmids. Actin blot was used as a loading control. The estimated size of EGFP-tagged Kv1.3 subunit with the FLAG epitope is between 85-95 kDa. The size of actin is 42 kDa. The marker was ProSieve QuadColor protein marker (4.6 kDa – 300 kDa**).**

Supplementary figure 4. Current of H399K truncated channels in CHO cells. a) Typical whole-cell current trace of FLAG-H399K-ΔC was recorded in a CHO cell upon 2-second-long, +50 mV depolarization from a holding potential of -120 mV. **b)** Whole-cell current recorded in a CHO cells expressing FLAG-H399K-NOHRET upon a depolarization for 200 ms to 0 mV from -120 mV holding potential.

Supplementary figure 5. Deletion mutants of Kv1.3 mutants targeting to the cell membrane of HEK cells. Representative images of EGFP- and FLAG-tagged Kv1.3 mutant channels expressed in HEK. ***a)*** HEK cells transfected with the A413V-FL, b) A413V-ΔC or ***c)*** A413V-NOHRET plasmid is shown. The 1st column shows the EGFP signal of Kv1.3 channel in green. Cells were labeled with anti-FLAG primary and Alexa Fluor 647 GAMIG secondary antibodies to verify cell membrane localiztion of the channel protein (2nd column, red). The 3rd column shows the merge of red and green channels of the EGFP and anti-FLAG fluorescence signal whereas brightfield images of the cells are shown in the 4th column. (For details of fluorescent labeling see *Materials and Methods*.) Scale bar is 5 μm.

Supplementary figure 6. Membrane-targeted fraction of truncated channels. We used the *watershed segment.p* package for Octave program[2] to measure the expression of the truncated and full-length mutant channels (A413V, H399K) at the plasma membrane and in the cytosol (for reference see http://peternagy.webs.com/image-analysis-with-matlab). The membrane-targeted fraction (MTF) was calculated using the following equation:

$$MTF = \frac{EGFP\ intensity\ in\ the\ membrane}{EGFP\ intensity\ in\ the\ membrane + EGFP\ intensity\ in\ the\ cytosol}$$

The EGFP membrane signal was determined using the FLAG labeling as a mask. The p values show versus-control (full-length phenotypes vs truncated channels) comparison for both point mutants. The p values for all pairwise analysis were 0.388 for H399K and 0.114 for A413V, when NOHRET and ΔC were compared. Mean ± SEM is shown for n≥8 cells.

Supplementary figure 7. Single channel conductance of HRETE mutant channels. Single channel current recordings in outside-out patch configuration from a CHO cells transfected with WT **(A)** and WT-XHRETE **(B)** plasmids. Patches were held at -40 mV then depolarized to 0 mV for 10 s. **(C)** Average single channel current measured at 0 mV for the WT(black bar), WT-XHRETE (gray bar), WT-polyA (dark gray column) and WT-Atail (light gray column), error bar represents SEM.

Supplementary figure 8. Targeting of WT-Atail channels to the cell membrane in CHO cells. Representative images of EGFP- and FLAG-tagged WT-Atail Kv1.3 mutant channels expressed in CHO. The EGFP signal of Kv1.3 channel in green, anti-FLAG primary and Alexa Fluor 647 GAMIG secondary antibodies is shown in red. The 3rd image from left shows the fusion of red and green channels. Rightmost snapshot is the bright field image of the cells.

Supplementary figure 1

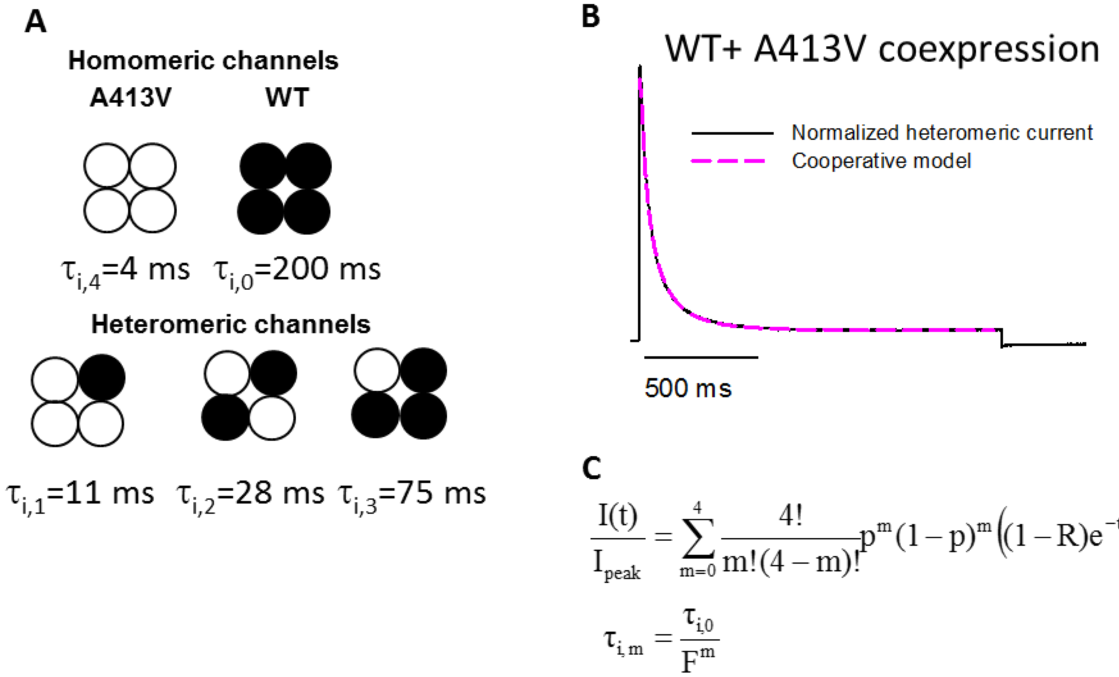

C

$$\frac{I(t)}{I_{peak}} = \sum_{m=0}^{4} \frac{4!}{m!(4-m)!} p^m (1-p)^m \left((1-R)e^{-t/\tau_{i,m}} + R\right)$$

$$\tau_{i,m} = \frac{\tau_{i,0}}{F^m}$$

Supplementary figure 2

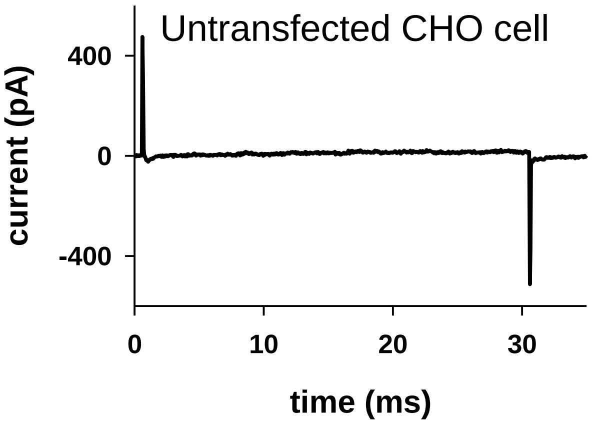

Supplementary figure 3

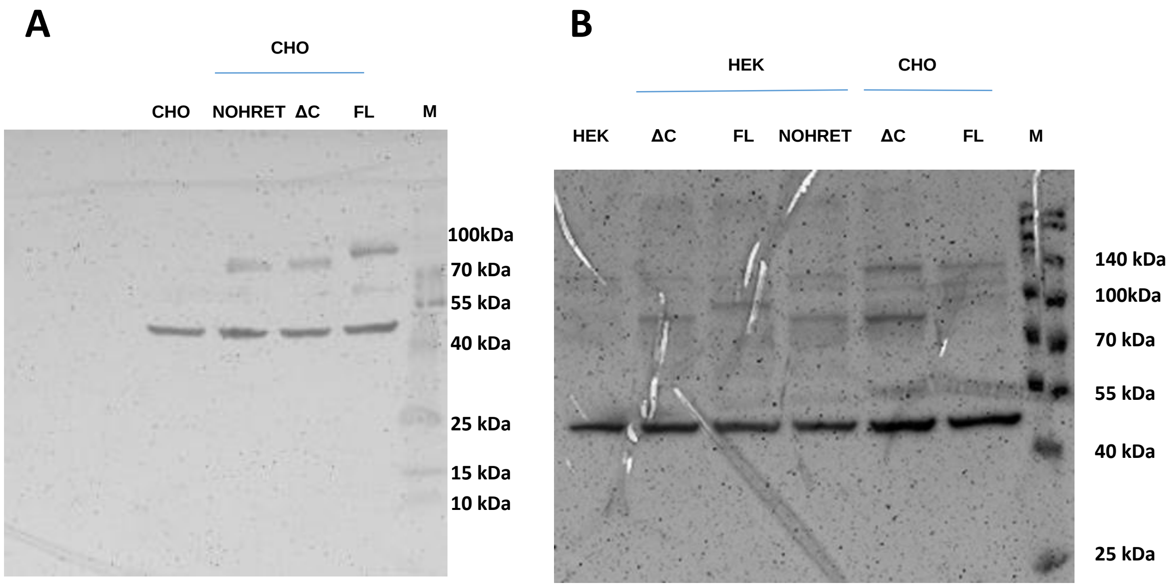

Supplementary figure 4

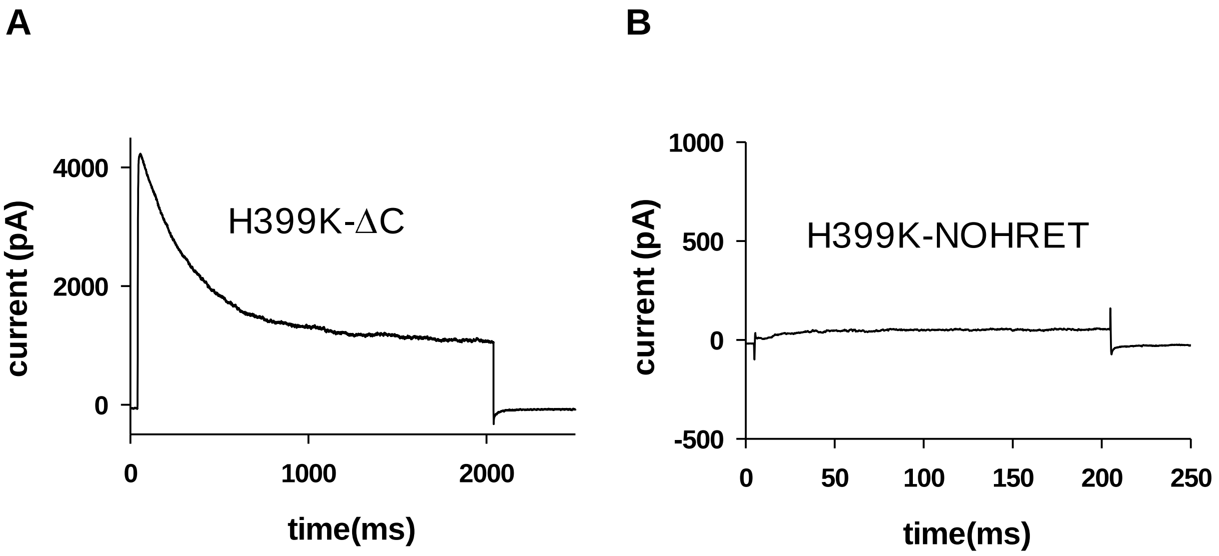

Supplementary figure 5

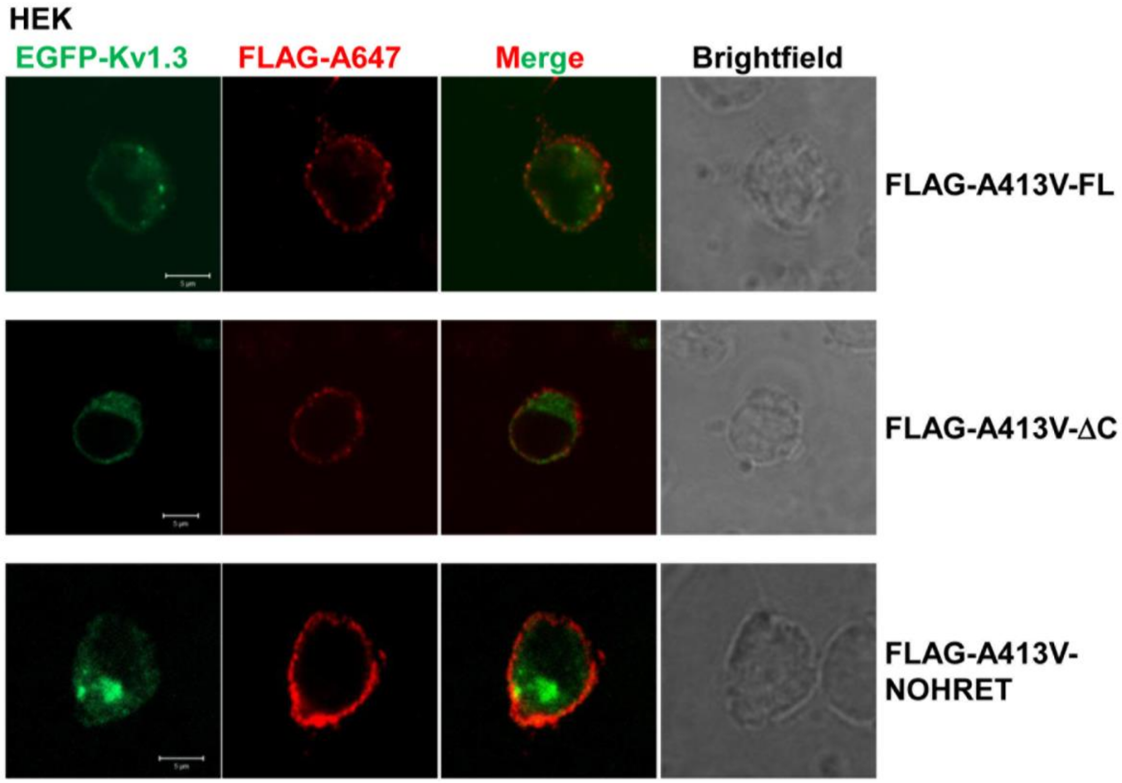

Supplementary figure 6

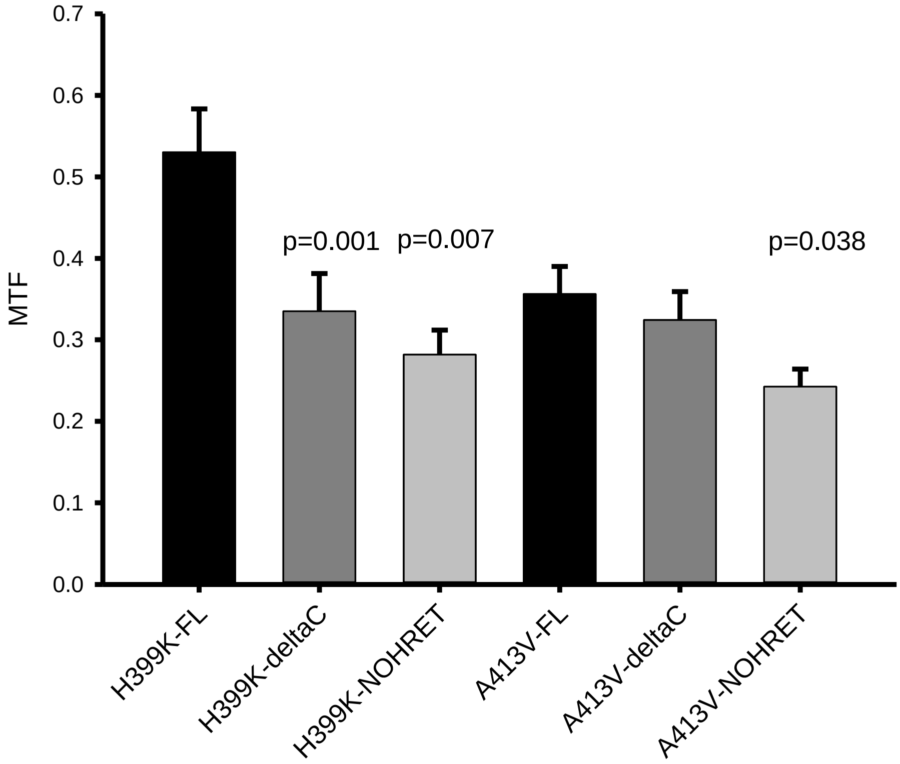

Supplementary figure 7

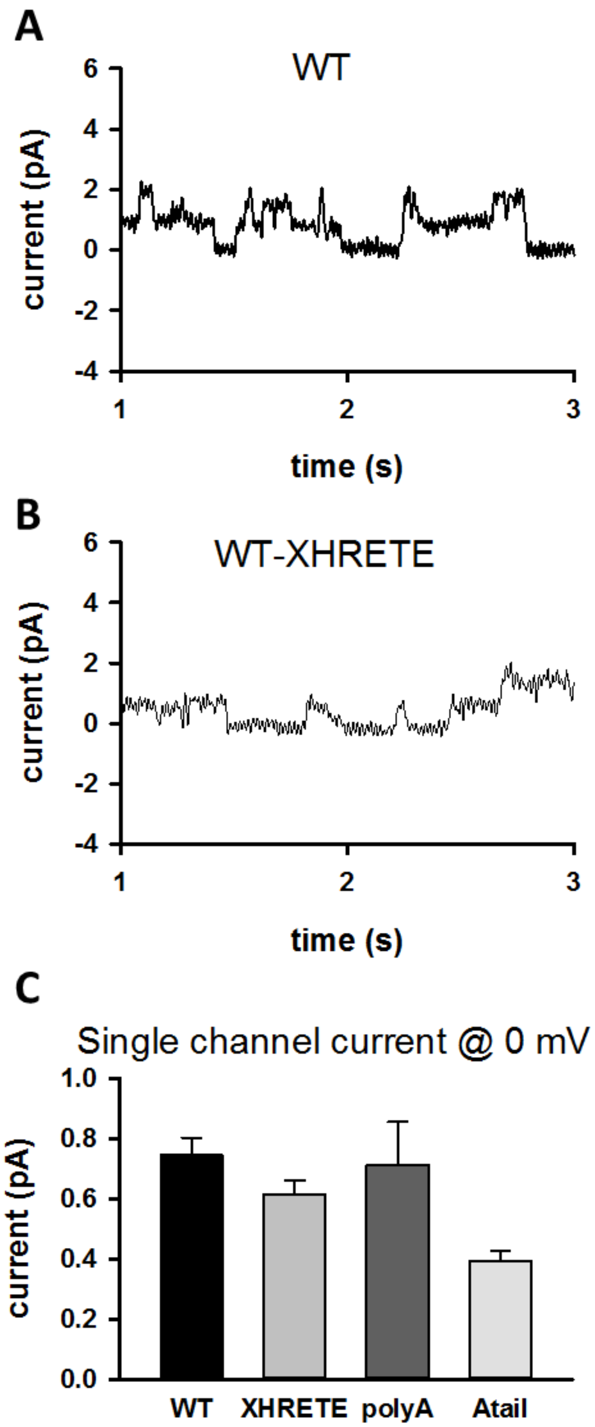

Supplementary figure 8

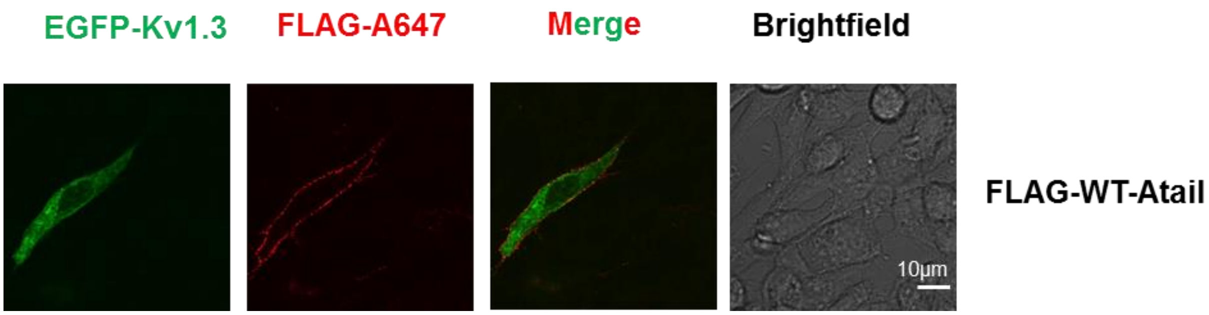

References

1. Panyi, G., Sheng, Z. & Deutsch, C. C-type inactivation of a voltage-gated K+ channel occurs by a cooperative mechanism. *Biophys J* **69**, 896-903, doi:10.1016/S0006-3495(95)79963-5 (1995).
2. Gonzalez, R. C., Woods, R. E. & Eddins, S. L. in *Digital Image Processing Using Matlab* (eds R.C. Gonzalez, R.E. Woods, & S.L. Eddins) Ch. 10.5, 417-425 (Pearson Prentice Hall, 2004).
